# Identification of the Rumination in Cattle Using Support Vector Machines with Motion-Sensitive Bolus Sensors

**DOI:** 10.3390/s19051165

**Published:** 2019-03-07

**Authors:** Andrew W. Hamilton, Chris Davison, Christos Tachtatzis, Ivan Andonovic, Craig Michie, Holly J. Ferguson, Laura Somerville, Nicholas N. Jonsson

**Affiliations:** 1 Department of Electronic and Electrical Engineering, University of Strathclyde, Glasgow G1 1RD, UK; christopher.davison@strath.ac.uk (C.D.); christos.tachtatzis@strath.ac.uk (C.T.); i.andonovic@strath.ac.uk (I.A.); c.michie@strath.ac.uk (C.M.); 2 Dairy Research and Innovation Centre, Scotland’s Rural College DG1 4TA, UK; holly.ferguson@sruc.ac.uk; 3 School of Veterinary Medicine, University of Glasgow, Glasgow G61 1QH, UK; somervillelm@gmail.com; 4 Biodiversity, Animal Health and Comparative Medicine, University of Glasgow, Glasgow G61 1QH, UK; nicholas.jonsson@glasgow.ac.uk

**Keywords:** cattle, bolus sensors, accelerometers, behaviour, rumination

## Abstract

The reticuloruminal function is central to the digestive efficiency in ruminants. For cattle, collar- and ear tag-based accelerometer monitors have been developed to assess the time spent ruminating on an individual animal. Cattle that are ill feed less and so ruminate less, thus, the estimation of the time spent ruminating provides insights into the health of individual animals. pH boluses directly provide information on the reticuloruminal function within the rumen and extended (three hours or more) periods during which the ruminal pH value remains below 5.6 is an indicator that dysfunction and poor welfare are likely. Accelerometers, incorporated into the pH boluses, have been used to indicate changes in behaviour patterns (high/low activity), utilised to detect the onset of oestrus. The paper demonstrates for the first time that by processing the reticuloruminal motion, it is possible to recover rumination periods. Reticuloruminal motion energy and the time between reticuloruminal contractions are used as inputs to a Support Vector Machine (SVM) to identify rumination periods with an overall accuracy of 86.1%, corroborated by neck mounted rumination collars.

## 1. Introduction

Dairy farmers are facing intense commercial pressure to optimize operational efficiency, which has driven the consolidation of farms and the application of technologies to increase milk production from fewer cattle. In the UK, the number of dairy producers has dropped from 35,741 in 1995 to 13,355 in 2015 and the number of dairy cows has fallen from 3.2 million in 1980 to 2 million in 2015 [1]. Despite these trends, the overall milk production in the UK has remained relatively constant or increased (13,319 million litres in 2008 versus 14,829 million litres in 2015) [2]. Operational scale and genetic gain are responsible in large part for this improved efficiency, but the implementation of different agritech sensing systems has given farmers greater insights into cattle fertility and health which, in turn, has increased milk yields [3,4,5,6]. This is underlined by the growth in sales of collars and pedometers for the detection of oestrus (or “heat”) to optimise herd reproductive efficiency [7,8,9,10,11]. Building on the commercial success of these devices, the industry is vigorously addressing the challenges of using technology to deliver other animal welfare-related information.

A key indicator of cattle health is the time spent ruminating, in which the animal regurgitates and masticates previously consumed food to aid digestion and nutrient absorption [12,13,14,15]. Cattle that are ill feed less consequently ruminate less, thus, the estimation of the time spent ruminating provides insights on the health of individual animals. A range of methods using microphones, accelerometers and laser interferometry have been evaluated to derive this information [16,17,18,19,20,21].

Directly monitoring the rumen pH value has been proven to be of value as it provides a strong indication of the onset of sub-acute rumen acidosis (SARA) [22,23,24,25]. Current definitions of SARA based on the pH of ruminal fluid rely on samples obtained using a stomach tube or by rumenocentesis. A cumulative period of more than 3 hours per day during which the ruminal pH values remain less than 5.6 is considered as being sufficient for diagnosis [22]. Other studies have suggested a single instance of rumen pH falling below a threshold of 5.5 as being a more appropriate measure [23]. 

Intubating animals or using rumenocentesis for routine rumen fluid extraction is labour-intensive and not carried out often. However, the commercial availability of reticuloruminal pH boluses that continuously relay pH information wirelessly to a central processing location has simplified the monitoring process and several commercial systems are currently available that deliver this function [26,27]. However, their wide-scale deployment remains constrained by the relatively short lifespan of the pH sensors. Several authors have reported sensors with excellent pH sensitivity and low power consumption that would support long term operation; however, the sensor lifetime is presently not limited by power consumption but by fouling due to rumen contents [28,29,30]. To date, no commercial system has an operational lifetime of more than a few months. 

While pH measuring has challenges, temperature sensors can be delivered in reticuloruminal bolus devices and these have operational lifetimes of several years. Despite being influenced by transient effects (such as drinking), the rumen temperature provides a good indicator of pyrexia. More recently, some manufacturers have integrated accelerometers into boluses to detect the onset of oestrus based on the increased overall activity by the cow [27]. The accurate detection of the onset of oestrus is core to efficient milk production. Failure to inseminate at the correct time will result in failure to conceive or the potential termination of pregnancy, leading to a loss of revenue. An accelerometer measures the overall activity of the animal and a significant increase in activity is a flag for the onset of oestrus. 

The rate and amplitude of ruminal contractions are influenced by metabolic diseases as well as any of the many diseases that cause pain or fever [22]. In addition to anorexia, clinical signs of indigestion include reticuloruminal hypo-motility [23]. In one study of 40 buffalos with histories of indigestion and inappetence, the reticuloruminal motility and rumination of every animal had decreased [24]. Reticuloruminal acidosis has also been shown to reduce the frequency of ruminal contraction, eventually resulting in ruminal stasis [24]. Hence, the measurement of reticuloruminal motility in its own right has value in the management of cattle to improve health, welfare and production performance. This study proves the potential for an accelerometer-based bolus to achieve this goal.

If reticuloruminal motion can be related to periods of rumination, then this approach provides a route identifying several welfare parameters—the overall activity, rumination, rumen motility and temperature—using a single device that senses the acceleration and temperature. In the present study, the focus is on the measurement and estimation of rumination time budgets by monitoring reticuloruminal contractions. To the authors’ knowledge, this has not been reported to date. Nogami et al. describe the integration of accelerometers within a bolus but have not related the measurements to feeding or rumination events in a rigorous manner [31]. The paper demonstrates that this is possible, thus enabling a single measurement instrument to provide an indication of rumination time (related to oestrus, welfare and calving), feeding time (related to oestrus and welfare) and temperature (related to welfare). 

## 2. Materials and Methods

### 2.1. Reticuloruminal Motion Monitoring

**Figure 1:**
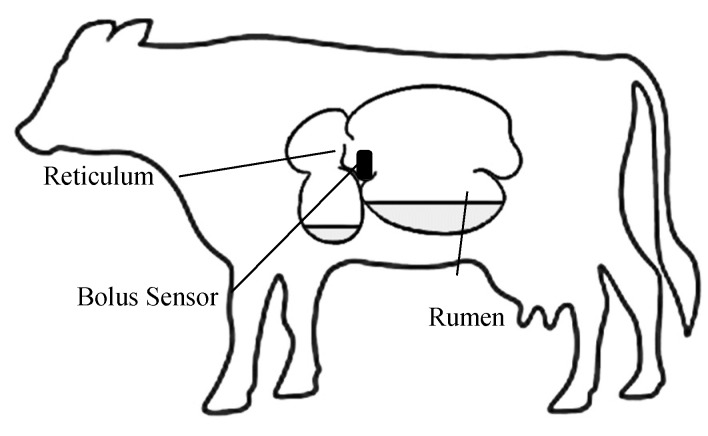
The simplified bovine digestive system and location of the bolus sensor.

Cattle have a large and complex stomach of four chambers executing on the alloenzymatic (enzymes provided by microbes resident in the stomach rather than enzymes produced by the host animal) digestion of cellulose-dominated complex carbohydrates. The first (proximal) compartment is the reticulum (Figure 1), which separates particulate matter from pure liquid and tends to act like a sump collecting heavy foreign bodies that have been swallowed. Consequently, the reticulum is the usual resting place of boluses that are administered by mouth [32]. The second compartment is the rumen, which is the largest chamber and major contributor to fermentation and digestion of organic matter. The two chambers (also referred to collectively as the reticulorumen) contract periodically in a dynamic relationship in order to pass fluid and material from one to the other and also to regurgitate food into the mouth for rumination (“chewing the cud”), an integral part of the digestion process. These rumination contractions are regular and typically occur at 40–60 second intervals. The objective of the study was to use an accelerometer-based bolus sensor within the reticulum to identify rumination periods from reticuloruminal motion. Accelerometer based motion sensors have been extensively researched for oestrus detection. Collar-based sensors, now routinely used in dairy farming, can identify rumination by monitoring accelerations on the neck muscles associated with the characteristic rhythmic jaw motion [17,18,19,33,34,35,36,37]. It is not possible to directly measure jaw motion using a reticuloruminal bolus, but reticuloruminal contractions have the potential to be an alternative route for the identification of rumination periods. However, the reticuloruminal motion is continuous and does not only occur during rumination; hence, a means of differentiating rumination periods from other motion is required.

### 2.2. Data Collection

**Figure 2:**
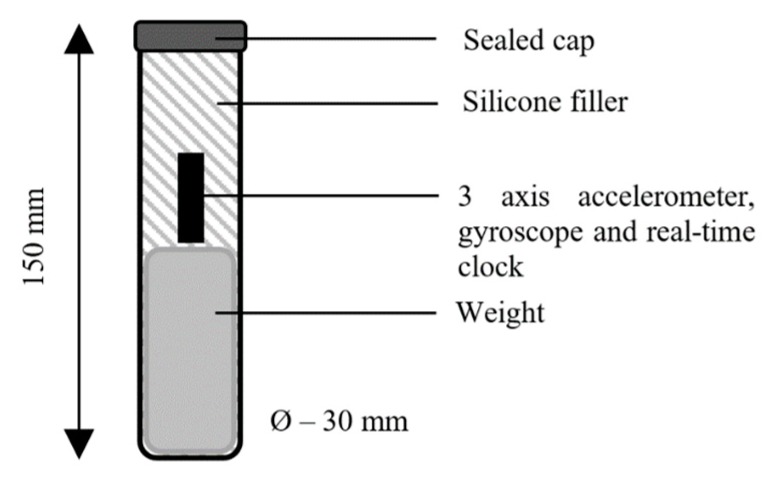
The layout of the bolus sensors used to monitor motion in the reticulum.

Three Jersey cows with ruminal cannulae were used to gather reticuloruminal motion data. Experimental studies on animals were conducted by holders of UK Home Office Personal Licences in compliance with the UK Home Office Project Licence 70/8600 (issued 17 August 2015). The cattle were housed indoors in a straw yard and fed hay ad libitum with 300 g of roller-milled barley grain fed to each cow at 08:00 and 15:00 each day. Each cow was equipped with a reticuloruminal bolus containing a 3-axis accelerometer, an SD card, and a Real Time Clock (RTC) together with a collar comprising of a 3-axis accelerometer, an SD card, and an RTC to gather feeding and rumination patterns from neck motions. Each bolus sensor was weighted by 250 g to ensure that it remained in close proximity to the floor of the reticulum in order to optimise the response to contractions in this chamber. The internal layout of the sensor and the accelerometer is illustrated in Figure 2. The sampling frequency for accelerometers was configured to be $f_{s}$ = 12.5 Hz. Neck-mounted accelerometer collars were used to monitor all animals to identify periods where rumination was taking place.

## 3. Data Processing and Classification

**Figure 3:**
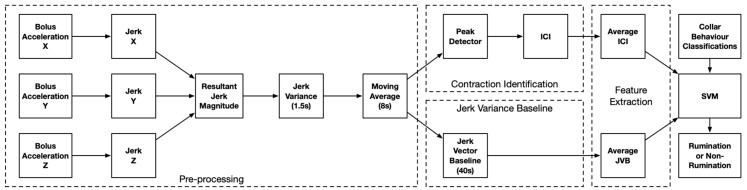
The overview of the processing of the bolus accelerometer data.

The objective was to extract features from the raw bolus accelerometer data which classify periods of rumination and verify these using the accelerometer collars. The boluses were collected after the study and the data were extracted from the internal storage (see Supplementary Materials section link to data). The data from the benchmark collars were also collected and a single dataset with uniform timestamps (from the respective real-time clocks) was created for each animal. The processing pipeline of the bolus accelerometer signals is shown in Figure 3, comprising pre-processing, aggregation and feature extraction. The extracted features from the bolus and the collar classifications were used to create a supervised Machine Learning (ML) model. Multiple ML approaches could be used for the classification of rumination and non-rumination periods such as Random Forest (RF), Neural Networks (NN) or Support Vector Machines (SVMs) [38]. In this study, an SVM was selected due to the low computational complexity and low memory requirements (a practical constraint of distributed sensors) to yield a prediction. Unlike RFs which create multiple decision trees and, consequently, a high number of parameters due to dichotomies, and NNs which contain multiple weights, SVMs require 2 hyper-parameters to execute the classification. The reduced complexity is desirable when considering the implementation of the classification methodology in terms of memory use, power consumption and constrained computational resources. 

### 3.1. Data Pre-Processing

**Figure 4:**
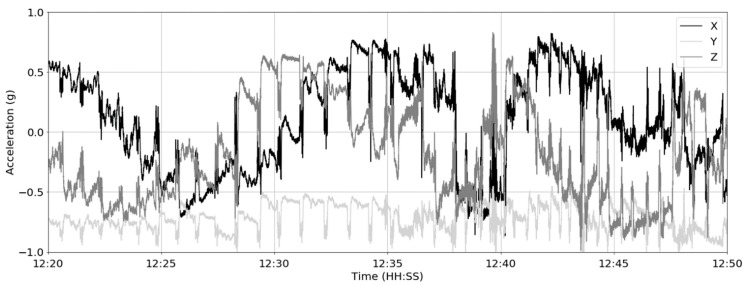
The x, y and z bolus acceleration for a 30 min period from cow #2.

The bolus sensors provided 3 acceleration measurements at a 12.5 Hz sampling frequency. A 30 min sample of the data from cow #2 is shown in Figure 4.

**Figure 5:**
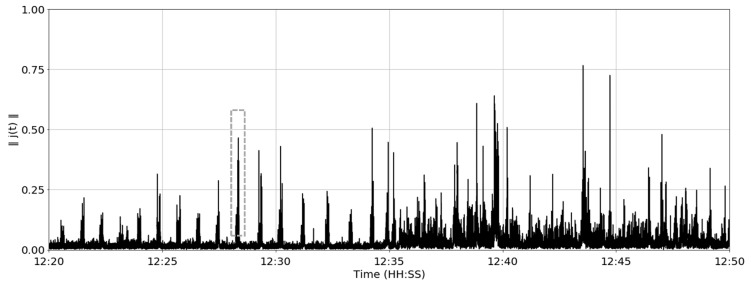
The magnitude of the resultant jerk vector over time for cow #2. Single peak during the apparent rumination period is illustrated with the grey dashed line.

Significant changes are observed in the bolus orientation despite the fact that it was weighted to maintain approximately vertical orientation within the reticulum as a consequence of the strength of the contractions. To eliminate steady-state orientation offsets and measure kinematic features solely attributed to the contractions, the individual axis acceleration values were resolved into a jerk as follows:

(1) $$j_{x}\left(t\right)=x\left(t\right)-x\left(t-\frac{1}{f_{s}}\right)$$

where *j_x_* is the jerk (i.e., the rate of change of acceleration) for the *x*-axis, *x*(*t*) is the acceleration at time *t* and *f_s_* is the sampling frequency (12.5 Hz). Resolving all three different jerk components into a single vector provides a measure of the resultant jerk magnitude:

(2) $$\|j\left(t\right)\|=\sqrt{{j_{x}}^{2}\left(t\right)+{j_{y}}^{2}\left(t\right)+{j_{z}}^{2}\left(t\right)}$$

where ‖*j*(*t*)‖ is the magnitude of the resultant (3D) jerk vector shown in Figure 5.

**Figure 6:**
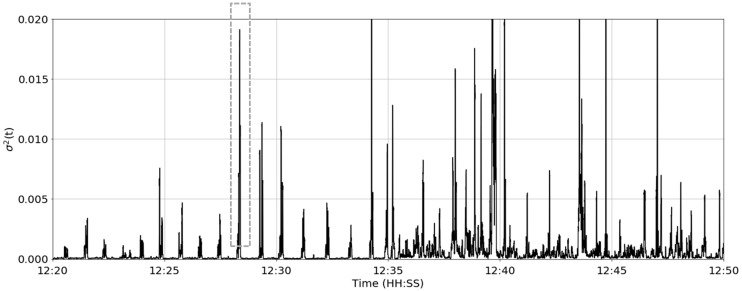
The rolling variance over a 1.5 s window for the same time period in cow #2. The grey dashed line indicates a single apparent rumination contraction with a duration of ≈8 s.

It can be observed in Figure 5 that the signal during the first 15 min is relatively low in magnitude with spikes approximately 50–60 s apart. In the latter 15 min, the underlying signal magnitude is higher with more frequent (but less periodic) spikes. These two states were consistently observed across all 3 cows during the total study. The hypothesis is that the first 15 min may indicate rumination periods since the peaks in the jerk magnitude are compatible with feed bolus regurgitation during rumination, whereas the last 15 min refers to eating or other higher energy activity. The duration of the contractions/regurgitation is approximately 8–10 s followed by a 40–50 s period where the bolus is subject to minimal movement whilst the cow is chewing. During the contraction periods, the signal has high variability while the variability is significantly reduced during the chewing periods. In order to accentuate the difference between these two periods, the rolling variance of the resultant jerk magnitude was evaluated over a 1.5 s rolling window:

(3) $$\sigma^{2}\left(t\right)=\sum_{n=0}^{w}\frac{{\left[\|j\left(t-n\cdot \frac{1}{f_{s}}\right)\|-\bar{\left\|j\left(t\right)\right\|}\right]}^{2}}{w}$$

where *σ*^2^ is the variance of the resultant jerk magnitude and *w* is the rolling window length. The rolling variance of the resultant jerk magnitude signal is shown in Figure 6.

**Figure 7:**
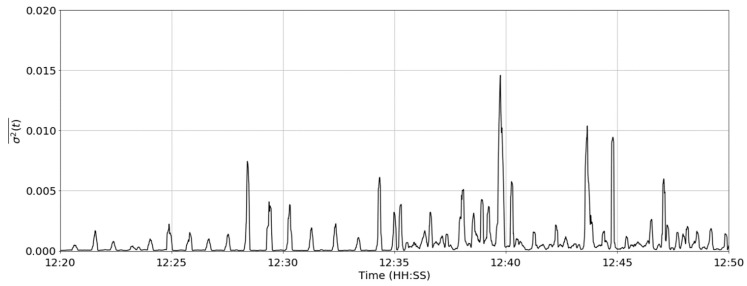
The mean of rolling variance over using an 8 s window.

The signal was subsequently smoothed using a rolling mean filter to reduce abrupt signal changes:

(4) $$\bar{\sigma^{2}\left(t\right)}=\sum_{n=0}^{w^{\prime }}\frac{\sigma^{2}\left(t-n\cdot \frac{1}{f_{s}}\right)}{w^{\prime }}$$

where *w*′ is the signal length. The value of *w*′ of 8 s was selected to approximate the duration of a single reticuloruminal contraction and the result is shown in Figure 7.

### 3.2. Contraction Identification and Inter-Contraction Interval

**Figure 8:**
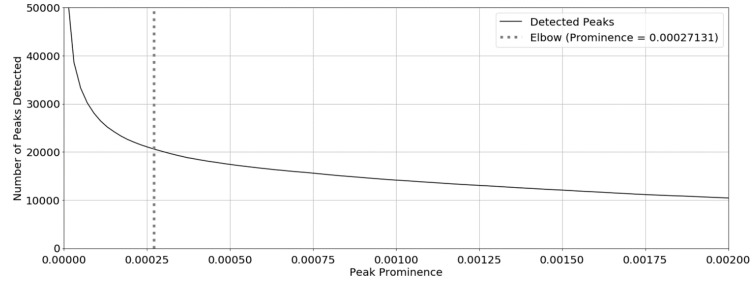
The sensitivity analysis of the peak prominence filter. The black trace is the number of detected peaks against prominence. The dashed vertical line corresponds to the location of the elbow determined using the technique in Reference [39].

**Figure 9:**
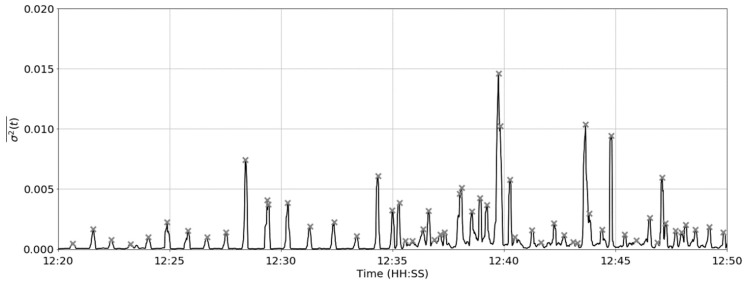
The peak detection with a prominence value of 0.00027.

A zero-derivative detector was used to identify the peaks in the trace. This approach is, however, vulnerable to error because any point of inflexion will be detected as a peak. To address this, minor inflexion points were discarded on the basis of their prominence, defined as the height of the peak relative to the lowest contour line. To identify a suitable prominence threshold, a sensitivity analysis on the number of peaks detected was performed and is shown in Figure 8. The prominence threshold was selected to be at the ‘elbow’ of the curve by using the method described in Reference [39]. Using this technique, the prominence threshold was found to be 0.00027. The detected peaks are shown in Figure 9.

Using the peaks, it is possible to determine the putative Inter-Contraction Interval (ICI) by computing the time difference between the peak locations.

### 3.3. Jerk Variance Baseline

**Figure 10:**
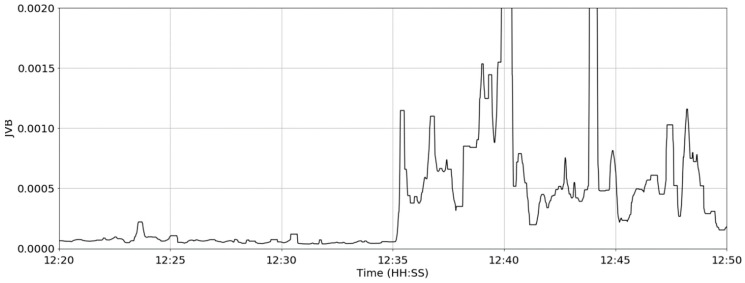
The Jerk Variance Baseline (JVB) signal.

The second feature of interest is a measure of the underlying signal energy calculated using a rolling median filter.

(5) $$JVB\left(t\right)=Md\left(\left\{\bar{\sigma^{2}\left(t-n\cdot \frac{1}{f_{s}}\right)},\;\forall n\left[0,w^{\prime \prime }\right]\right\}\right)$$

where Md(·) denotes the median and *w*″ is the rolling window length (40 s)—approximately one inter-contraction period. The output of the filter (herein, referred to as the Jerk Variance Baseline (JVB)) is shown in Figure 10. 

### 3.4. Feature Extraction

**Figure 11:**
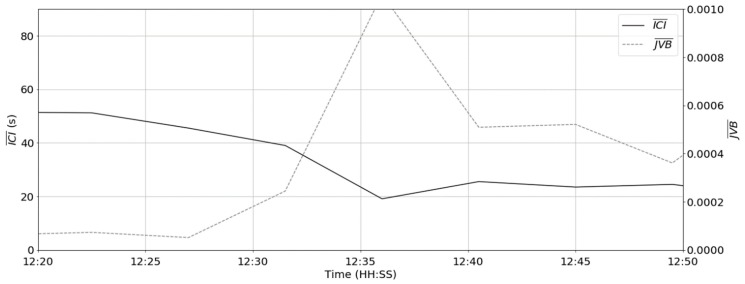
The comparison of the block (270 s) Inter-Contraction Interval (ICI) and JVB over time.

Computing the average ICI and JVB statistics over a period of 270 s resulted in the trace shown in Figure 11. A block size of 270 s was selected to capture a sufficient number of peaks to gain the representative ICI/JVB values. It can be observed that the average ICI and JVB reflect the change in the apparent state over time. 

### 3.5. Support Vector Machine Classification using ICI and JVB Features

From visual observation across the dataset, it was postulated that the periods characterised by ICI around 40–50 s and a low JVB were periods of rumination. ICI and JVB were used as inputs to a Support Vector Machine. Truthing data (rumination periods), identified using neck mounted activity collars, were used to direct the supervised learning process.

The activity collars classified rumination, feeding and other states in 90 second intervals. This time interval was too short to obtain an average value of ICI. Therefore, the collar readings were aggregated into 270 s periods. Boluses with corresponding collar data for the three cows were used, resulting in a balanced dataset with 2424 periods of measurement and observation data.

**Figure 12:**
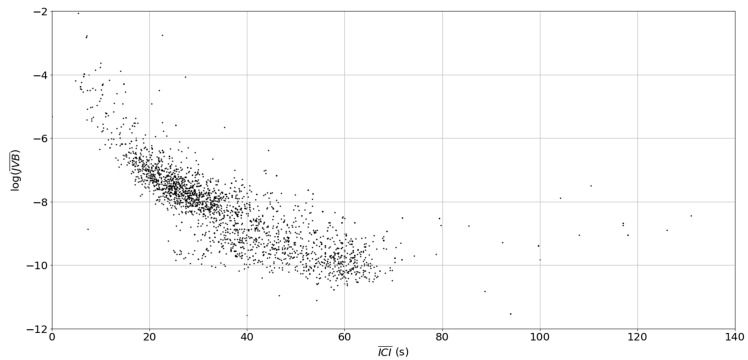
The block ICI vs. log(JVB), each point represents a 270 s block window.

The scatter plot of $\bar{ICI}$ vs. log($\bar{JVB}$) is shown in Figure 12. There is a dense cluster with an approximate centroid at $\bar{ICI}$ = 25 s and log($\bar{JVB}$) = −7.8 with another cluster with an approximate cluster at $\bar{ICI}$ = 45 s and log(JVB) at −9.9. 

To train the Support Vector Machine, the dataset was split by a ratio of 80/20 for training/testing respectively using random sampling and with each class balanced (rumination or non-rumination) to prevent a disproportionate number of data points with a certain class from being selected. 

A linear SVM was used and verified using k-fold cross-validation to determine the robustness of the prediction. The training data was split into a further 10 sets (or folds) where each set contained a unique combination of data points, meaning that each set did not contain any duplicates, allowing for the assessment of the impact on random data selection through the potential variations on the decision boundary. The optimal number of folds is dependent on the dataset; 10 has been shown to offer a good compromise between balancing variance reduction and sample bias.

## 4. Results

To assess the performance of the features ($\bar{ICI}$ and $\bar{JVB}$), it was necessary to evaluate the prediction accuracy of the model and compare that to the collar classification.

### 4.1. Linear SVM

**Figure 13:**
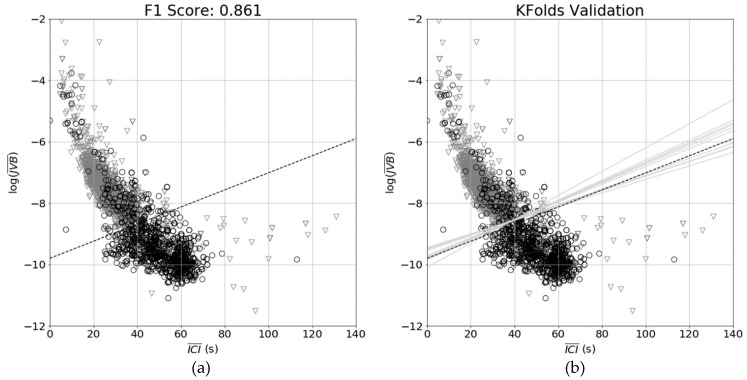
The Support Vector Machine (SVM) model: (**a**) the linear SVM decision boundary indicated by the black dashed line with the collar classification of rumination for each point (BLACK CIRCLES: rumination, GREY TRIANGLES: non-rumination); (**b**) the decision boundary for each of the folds shown by light grey lines.

In Figure 13a, the result of the linear SVM training and testing is shown. The classification of the collar is shown by colour, with black circles indicated a period of rumination, and grey triangles indicated non-rumination. The decision boundary (shown by blacked dashed line) is the result of the linear SVM model fitting on the training data. The F1 score was used to evaluate system performance, defined as the geometric mean of precision and recall defined as

(6) $$F1=2\cdot \frac{precision\cdot recall}{precision+recall}$$

where *precision* is defined as

(7) $$precision=\frac{True\;Positives}{True\;Positives+False\;Positives}$$

where a True Positive (TP) represents an instance where the rumination had correctly been identified. A False Positive (FP) is a prediction of rumination during a period where the cow was not ruminating. *Recall* represents the percentage of actual rumination events that were correctly identified:

(8) $$recall=\frac{True\;Positives}{True\;Positives+False\;Negatives}$$

where a False Negative (FN) identifies a case where a period of rumination was incorrectly judged to be ‘not ruminating’. To evaluate the model’s ability to detect non-rumination, the *specificity* and *negative predictive value* were used:

(9) $$specificity=\frac{True\;Negatives}{True\;Negatives+False\;Positives}$$

(10) $$negative\;predictive\;value=\frac{True\;Negatives}{False\;Negatives+True\;Negatives}$$

In Table 1, the confusion matrix for the SVM model with the F1 score found to be 0.861 on the test data is shown; 2424 samples were used to train and validate the model.

### 4.2. k-Folds Validation

The results of the k-folds validation are shown in Figure 13. Each of the light grey lines represents the decision boundary derived from each of the splits in the training data, while the final model is shown by the dashed black line. It can be observed that there is a relatively consistent boundary, with the F1 scores, precision and recall shown in Table 2, indicating that the SVM model was not adversely affected by sample selection.

## 5. Discussion

Motion from an accelerometer within a rumen bolus has been analysed using a linear SVM model to classify periods of rumination in cattle. Using a 10-fold validation, the performance produced an average F1 score of 0.861 with a variance of 0.000591, indicating that the model was not significantly affected by selection bias. A precision of 0.832 and a recall of 0.892 indicate that the model is able to detect rumination periods accurately whilst the negative predictive value of 0.928 and a specificity of 0.888 shows that the model also performed well at identifying periods of non-rumination. The selection of a linear SVM allowed a distinct decision boundary to be estimated. Other SVM models (e.g., polynomial) were examined, however, the performance was significantly affected by outliers. Classification of rumination using activity collars, halters and ear tags has been described in Reference [40], where the F1 score for rumination prediction was reported. Halters were found to have the best performance (F1 = 0.932) due to the sensor directly measuring jaw motion through pressure within a muzzle. Collars were evaluated with an F1 score of 0.913 and ear tags with 0.895, as a consequence of the reduced Signal-to-Noise ratio. In this current study, the bolus and SVM classification performed comparably with an F1 of 0.861. The classification of rumination using a bolus with the SVM model represents significant progress because it enables rumination patterns to be identified using a transducer located within the animal, considerably increasing the potential to remotely identify welfare-impairing conditions in cattle. The system has the advantage in that it can be administered during the routine deployment of boluses, carried out to deliver slow release nutrient supplements and will be retained for the lifetime of the animal. This eliminates the potential for sensory loss, a known issue with ear tags, and the potential for animal strangulation or physical damage to the sensor which can occur with collar-based systems.

## 6. Conclusions

The reticuloruminal function is a key element of the digestive efficiency in ruminants and the automated measurement of this central process provides valuable insight into animal welfare. This study aimed to establish whether or not the act of ruminating could be identified through measurements of reticuloruminal motion. Reticuloruminal contractions occur continuously irrespective of whether an animal is ruminating or not. Therefore, a mechanism for identifying rumination periods from other behaviours is required. In the study, this was addressed using a Support Vector Machine trained using neck mounted collars achieving an F1 score of 0.861. To our knowledge, this is the first time this has been reported. Diagnosing rumination behaviour using a rumen mobility sensor paves the way to produce other diagnostic measurements from the strength and amplitude of the rumen contraction, representing, to our knowledge, the first time that this has been reported.

## Figures and Tables

**Table 1:** The confusion matrix for the classification of rumination by the Support Vector Machine (SVM) model with collar validation.

		**True Rumination Class**		
		**Rumination**	**Non-rumination**	
**Predicted Rumination Class**	**Rumination**	TP = 34.3%	FP = 6.9%	Precision = 0.832
	**Non-rumination**	FN = 4.2%	TN = 54.6%	Negative Predictive Value = 0.928
		Recall = 0.892	Specificity = 0.888	F1 = 0.861

**Table 2:** The F1 scores of the k-folds for validation.

Fold #	1	2	3	4	5	6	7	8	9	10
F1 Score	0.871	0.916	0.844	0.844	0.851	0.855	0.803	0.821	0.823	0.839
Precision	0.853	0.893	0.792	0.810	0.836	0.801	0.790	0.833	0.817	0.839
Recall	0.889	0.941	0.903	0.882	0.866	0.917	0.816	0.810	0.830	0.840

## Supplementary Materials

All data underpinning this publication are openly available from the University of Strathclyde at: https://doi.org/10.15129/fc809102-7288-4b4e-aa48-55ba6608dc2c.
